# Association and interaction of the MC4R rs17782313 polymorphism with plasma ghrelin, GLP-1, cortisol, food intake and eating behaviors in overweight/obese Iranian adults

**DOI:** 10.1186/s12902-022-01129-w

**Published:** 2022-09-19

**Authors:** Sara Rahati, Mostafa Qorbani, Anoosh Naghavi, Hamideh Pishva

**Affiliations:** 1 Department of Cellular - Molecular Nutrition, School of Nutrition Sciences and Dietetics, Tehran University of Medical Sciences, PO Box: 14155-6447, Tehran, Iran; 2 Non-Communicable Diseases Research Center, Alborz University of Medical Sciences, Karaj, Iran; 3 Cellular and Molecular Research Center, Resistant Tuberculosis Institute and Department of Genetics, Zahedan University of Medical Sciences, Zahedan, Iran

**Keywords:** MC4R, Appetite, Food intake, Emotional eating, Stress, Anxiety, Depression

## Abstract

**Background:**

Recent studies have shown that obesity is largely influenced by heredity and created by the interactions between several genes and environmental and behavioral factors. This study aimed to examine association between variant rs17782313 near melanocortin-4 receptor (MC4R) gene and behavioral and hormonal factors then evaluated interactions between variant MC4R rs17782313 with behavioral and hormonal factors on obesity.

**Methods:**

This cross-sectional study included 403 subjects, overweight and/or obesity, aged 20–50 years from Iran. The MC4R rs17782313 data were measured by the PCR–RFLP method. Dietary intake, physical activity, stress, anxiety, depression, appetite and emotional eating were assessed by using validated questionnaires. Ghrelin, glucagon-like peptide-1 and cortisol were measured by radioimmunoassay in plasma samples. Participants were also divided into three groups based on rs17782313 genotype and BMI.

**Results:**

After adjustment for age, gender, energy intake and PA, significant associations were observed between food intake, appetite, emotional eating, stress and physical activity with MC4R rs17782313 (*p* ˂0.05). Also, significant interactions were observed between fat intake (*p-interaction* = 0.002), protein intake (*p-interaction* = 0.01), energy intake (*p-interaction* = 0.01), emotional eating (*p-interaction* = 0.02), appetite (*p-interaction* = 0.04), stress (*p-interaction* = 0.04), ghrelin (*p-interaction* = 0.03), cortisol (*p-interaction* = 0.04) and physical activity (*p-interaction* = 0.04) and MC4R rs17782313 in terms of BMI.

**Conclusion:**

Interactions between the CC genotype and high intakes of fat and energy, emotional eating, high appetite, and too much stress with high levels of cortisol and ghrelin probably can have an effect on BMI in overweight/obese subjects.

**Supplementary Information:**

The online version contains supplementary material available at 10.1186/s12902-022-01129-w.

## Introduction

Obesity has become a worldwide epidemic and still rising at an alarming rate. It can dramatically affect the quality of life and increase the risk of metabolic disorders including diabetes, hypertension, and cardiovascular complications [1]. Obesity is highly heritable and arises from the interactions of multiple genes, environmental and behavioral factors. Numerous hormonal and neurological factors regulate normal body weight. These factors are encoded by genes [2]. Minor variations in gene expression or interactions of these variations with environmental factors may result in obesity. Abnormalities in the circuits regulating energy homeostasis play a crucial role in weight gain. Variations in energy balance determine body composition and weight. Central nervous system (CNS) including central melanocortin pathway regulates energy balance [3]. Melanocortin-4 receptor (MC4R) is a critical regulator of energy homeostasis and appetite [4, 5]. Mutations in the MC4R gene is the most prevalent monogenic cause of obesity [6, 7], with a prevalence of 1.7–3.0% among obese people [8]. In this sense, the variant rs17782313 near the MC4R gene has been strongly linked with obesity [9, 10] and showed a significant association with dietary intake [11–13], total energy intake [14], increased snacking, as well as hunger [15].

Evidence suggested that brain melanocortinergic system especially MC4R has a well-established role in stress-induced changes, behavioral responses to stress and stress-related psychological disorders [16]. Glucocorticoids regulate body fat accumulation and storage. Glucocorticoids can also increase appetite, food intake and weight gain. Acute and chronic exposure to stress with increased cortisol can alter both the quantity and quality of calories consumed by humans. Stress-induced alterations in food intake and energy balance can interact with emotional state and appetite [17–19].

The glucagon-like peptide-1 (GLP-1) is a gut-brain hormone that coordinates several prandial and postprandial metabolic functions, including gastric emptying, incretin effect and satiation [20, 21]. The peripheral peptide hormone ghrelin is a powerful stimulator of food intake, which leads to body weight gain and adiposity. The hormone, thus, increases the vulnerability to obesity and binge eating behavior [22, 23]. Association of emotional eating with dietary habits and body weight is currently a widespread topic of discussion in studies related to obesity [24]. Studies have shown that emotionally sensitive people eat more food when they are dealing with negative emotions such as anger, irritability, fear, sadness or boredom. Therefore, emotional eating has a significant association with weight gain and increased intake of high-calorie foods with low nutritional value [25].

A limited number of studies have examined the association between MC4R rs17782313 polymorphism and obesity-related behaviors as well as the interaction of these factors with MC4R rs17782313 on obesity. Therefore, we decided to investigate the association of MC4R rs17782313 polymorphism with food intake, emotional eating, appetite, appetite hormones, physical activity, stress, and stress hormone. Furthermore, potential interactions between above factors and MC4R rs17782313 for obesity have been also tested. Considering that previous studies have identified this polymorphism as the most significant MC4R polymorphism associated with obesity, the research focused only on obese and overweight individuals.

## Materials and methods

### Participants

Two hundred twelve men and 191 women healthy overweight or obese (25 kg/m^2^ ˂ BMI ˂ 40 kg/m^2^) within the age range of 20–50 years were included in this cross-sectional study. The study population was collected from all regions of Zahedan, using community-based sampling and cluster sampling. Patients receiving thermogenic or lipogenic drugs, or those diagnosed with diabetes mellitus, chronic renal failure, hepatic diseases, hyperthyroidism, hypothyroidism or cancer were excluded from the study. All subjects were genotyped for the near MC4R rs17782313, using a Polymerase Chain Reaction-Restriction Fragment Length Polymorphism (PCR–RFLP) of their MC4R genotypes. The data were collected from June to October 2019. The study was approved by Ethics Committee of Tehran University of Medical Sciences (NO: IR.TUMS.VCR.REC.1398.260). All participants were informed of the study nature and gave written consents. The study was conducted at the Department of Cellular-Molecular Nutrition, TUMS.

### Sample collection, DNA extraction, and genotyping

At the beginning of this study, 10 cc blood was collected from each patient. Blood samples were collected after 12 h of overnight fasting in tubes with the anticoagulant, Ethylenediaminetetraacetic acid (EDTA). Genomic DNA was extracted from whole blood using the GeneAll, Exgene™Cell SV kit (Gene All, Korea) based on the constructor's protocol. DNA fragment containing a thymine-to-cytosine (T ˃ C) substitution in 188 kb downstream of MC4R gene was genotyped by PCR–RFLP as follows. The PCR amplification of the genomic DNA fragment for MC4R was performed by the forward primer 5′ AAG TTC TAC CTA CCA TGT TCT TGG 3′ and reverse primer 5′ TTC CCC CTG AAG CTT TTC TTG TCA TTT TGA T 3′ (ATCG company, Denmark) [26, 27]. The PCR was performed in a total 20 μl containing 0.3 μmol of each primer, 2 μg of DNA, 10 μl of Taq DNA Polymerase 2 × MasterMix (Ampliqon, Germany). The amplification protocol considered of an initial denaturation step at 95 °C for 2 min, followed by 35 cycles of denaturation at 95 °C for 30 s, annealing at 58 °C for 30 s, and extension at 72 °C for 30 s, and final extension at 72 °C for 5 min. Digestion was performed with 10.5 μl of each PCR product, incubated with 0.15 μl of BclI (10 U/μl, Fermentas, USA) and 1.5 μl of 10 × restriction G-buffer (in total 21.15 μl reaction) over-night at 56 °C and then the reaction was stopped at 0 °C for 5 min. The digested product was then subjected to electrophoresis on 2% agarose gel (Boehringer Manheim GmbH, Mannheim, Germany), stained with green viewer (Pars Tous, Iran) and visualized on a Gel Doc-system (U.V.P Company, Cambridge, UK). A 50 bp Ladder (Fermentas, Germany) was used to determine the Length of the digested products. The C allele appeared as a 137 bp fragment after electrophoresis, whereas the T allele was cleaved by the registration enzyme and appeared as 30 bp and 107 bp fragments. Finally, 10% of the samples were randomly selected and regenotyped to confirm the results.

### Ghrelin, GLP-1 and Cortisol

Blood samples were collected from all subjects in the morning, after 10 to 12 h of fasting and were centrifuged at 4 °C, and the plasma was stored at -80 °C for subsequent analysis. Plasma GLP-1 [28], ghrelin [29] and cortisol [30] were measured by radioimmunoassay (Linco Research, St. Charles, MO). All samples for GLP-1, ghrelin and cortisol were run in duplicate.

### General, anthropometric and physical activity assessments

We collected general information, such as age, educational level, marital status, and history of weight loss in recent years using standard questionnaires. Weight and height were measured using the Seca scale (GMbH, Hamburg, Germany) with light clothing and no shoes on. BMI was calculated as the weight in kilograms divided by the square of height in meters. Waist circumference (WC) was measured midway between the iliac crest and the lower costal margin along with hip circumference (HC) and waist-to-hip ratio (WHR) was calculated as WC/HC. All anthropometric measurements were taken in accordance with World Health Organization standards [31]. The official Persian short form of International Physical Activity Questionnaire (IPAQ-SF) was administered for evaluation of the physical activity level. This questionnaire was previously translated into Persian and validated in Iranian adult population [32]. The IPAQ was categorized as follows: low < 600, moderate (600–3500), and high (> 3500) Metabolic Equivalent of Task (MET)-h/wk [33].

### Dietary intake assessment

Dietary intakes were assessed by expert dietitians using a validated, 3-day food record [26]. The participants were trained by expert dietitians in how to complete the 3-day food record Questionnaire. The participants were also asked to record every detail and quantities of their consumer foods in six meals (breakfast, lunch, dinner, and three snacks) for three days (two weekdays and one weekend) in the questionnaire. After recording three days of their diets, they were supposed to visit the laboratory at the end of the week. The questionnaires were delivered and analyzed. Moreover, the participants providing insufficient or excessive reports were excluded from the study in the data analysis phase. Total energy and dietary nutrients were assessed by the Iranian Food Composition Table (FCT) and N4 software.

### Assessments of appetite

Visual analogue scales (VAS) of 100 mm in length were fill out for assessment of self-reported appetite sensations while fasting (satiety, fullness, hunger, and prospective foods consumption). An overall appetite suppression score was calculated based on four appetite parameters using the following formula (satiety + fullness + [100 – hunger] + [100 – prospective food consumption])/4, with 0 indicating higher appetite/less satiety and 100 indicating lower appetite/more satiety (primary assessment of the self-reported appetite sensations) [34].

### Emotional eating

The self-report Emotional Eating Questionnaire (EEQ) was used at the beginning of the program to assess emotional eating behavior. This tool has been directly developed and validated in a Spanish population consisting of overweight and obese people [35]. This 10-itme scale investigates the effects of emotions on eating behavior, and categorizes people into four groups based on their overall EEQ scores [25]. The validity and reliability of the questionnaire has been established prior to the present study. Cronbach ‘s α and ICC were 0.73 and 0.65–0.87, respectively.

### Stress assessment

Depression, anxiety, stress scale-21 (DASS-21) which is a validated questionnaire [36] was utilized to get the related scores on the amount of depression, anxiety and stress. Validation of the Persian model of this tool showed acceptable internal steadiness [37]. The short version has 21 items that are divided into 7 items each evaluating the signs of depression, anxiety and stress respectively.

### Statistical analysis

Statistical analysis was carried out by using SPSS software for Windows (version 26) (Chicago, IL, USA). The normality of variable distribution was tested by the Kolmogorov–Smirnov test. A chi-square test was used to assess Hardy–Weinberg equilibrium. Association between variables and MC4R rs17782313 genotypes was analyzed in different groups. One-way ANOVA and kruskal–wallis were used to compare continuous variables among different genotypes with normal and abnormal distribution, respectively.

The difference between distribution of macronutrients (adjust energy by residual method) and energy in study groups was calculated with regard to age and sex using analysis of covariance (ANCOVA) method according to 3 genotypic groups of MC4R rs17782313 after adjustment. The categorical variables were analyzed by using chi-square test. Any significance relationship between variables was further investigated with regression analysis.

Relationship between appetite factors, cortisol, food intakes and physical activity with different MC4R genotypes in study groups was determined using multivariate linear regression. The results of linear regression were presented as (β) coefficients and confidence intervals (95% CI). Also, the relationship between emotional eating, stress, anxiety and depression with different MC4R genotypes in study groups was performed using logistic regression. The results of logistic regression were presented as odds ratio (OR) and 95% CI. In regression analysis, in the crude model, the raw relationship (without adjust) of variables with genotypes was examined and in the first model, this relationship was adjusted for age, sex, and energy intake. In the second model, this relationship was adjusted for age, sex, energy intake, marital status, smoking status, occupation, education, and physical activity. To examine the interaction between the MC4R rs17782313 variants and behavioral and hormonal factors on obesity, logistic regression models were used for interaction terms in addition to the potential confounders. P-value ˂ 0.05 was considered statistically significant.

The present study examined research variables in three groups: overweight, obese, and the entire population (total overweight and obese) based on three genotypic groups: TT, CT, and CC. The goal of this grouping was to examine the separate relationships between overweight, obese, and the entire population and study variables.

## Results

The frequency of minor allele of MC4R is 44%. The distribution of MC4R rs17782313 genotypes (TT, TC, and CC) is not in Hardy–Weinberg equilibrium (*p* ˂ 0.001). The means and standard deviation (SD) of age, weight, BMI, and WC of total, overweight and obese subjects were (36.5 ± 8.7, 35.9 ± 9.3, 37.3 ± 7.8 years), (85.8 ± 10.6, 80.2 ± 7.8, 92.3 ± 9.2 kg), (30.2 ± 3.1, 28 ± 1.1, 32.8 ± 2.6 kg/m2), and (99.01 ± 8.8, 94.5 ± 7.1, 104.2 ± 7.6 cm), respectively.

### Baseline characteristics of overweight, obese and total papulation

The baseline characteristic of subjects according to MC4R rs17782313 genotypes is given in Table 1. Anthropometric variables and outcomes of study were compared in different MC4R rs17782313 genotypes. Analysis of variance was used to compare the means of these variables in MC4R rs17782313 genotypes. Significant differences were found in weight of all participants (*p* ˂0.001) and obese subjects (*p* = 0.008), waist circumference of individuals with obesity (*p* = 0.03) and all subjects (*p* = 0.001) and WHR of all participants (*p* = 0.02) between different genotypes of MC4R. However, no significant differences were found in other anthropometric variables between different genotypes of MC4R rs17782313. A statistically significant difference was found in physical activity of different genotypes of MC4R rs17782313 in individuals with obesity (*p* < 0.001) and all subjects (*p* < 0.001) (Table 1).

**Table 1:** Association of MC4R variant rs17782313 with baseline demographic characteristics

Variables	Overweight (*n* = 221)				Obese (*n* = 182)				Total (*n* = 403)			
	Genotype *P*-value^*^				Genotype *P*-value^*^				Genotype *P*-value^*^			
	TT(*n* = 71)	CT(*n* = 127)	CC(*n* = 23)		TT(*n* = 29)	CT(*n* = 123)	CC(*n* = 30)		TT(*n* = 100)	CT(*n* = 250)	CC(*n* = 53)	
Age(year)	37.4 ± 8.5	35.1 ± 9.4	35.2 ± 11	0.23	34.7 ± 8.6	37.2 ± 7.6	39.9 ± 7.2	0.04	36.9 ± 8.6	36.2 ± 8.6	38 ± 9.2	0.42
Weight(kg)	79.2 ± 7.3	81 ± 8.2	78.9 ± 6.8	0.20	89.9 ± 11^a^	91.7 ± 8	96.9 ± 9^a^	0.008	82.3 ± 10^ab^	86.4 ± 10^a^	89.5 ± 12^b^	<0.001
Males(%)	47.9(34)	63.8(81)	47.6(10)	0.06	34.5(10)	47.6(59)	63.3(19)	0.08	44(44)	55.6(140)	56.9(29)	0.12
Waist circumference(cm)	93.6 ± 7.6	95.4 ± 7	92 ± 5	0.05	102.7 ± 8^a^	103.8 ± 7	107.4 ± 8^a^	0.03	96.2 ± 8.8^a^	99.6 ± 8.3	101 ± 10^a^	0.001
Hip circumference(cm)	100.5 ± 9	101.7 ± 9	97.3 ± 7	0.11	108.6 ± 14	107.1 ± 7	107.4 ± 8	0.73	102.9 ± 11	104.4 ± 9	103.2 ± 9	0.34
Waist/Hip Ratio	0.92 ± 0.1	0.93 ± 0.1	0.94 ± 0.0	0.50	0.95 ± 0.1	0.96 ± 0.0	0.99 ± 0.0	0.19	0.93 ± 0.1^a^	0.95 ± 0.0	0.97 ± 0.0^a^	0.02
Married(%)	80.3(57)	73.2(93)	61.9(13)	0.22	75.9(22)	85.5(106)	83.3(25)	0.45	79(79)	79.4(200)	74.5(38)	0.44
Physical activity (MET-h/week)	32.4 ± 14^a^	23.7 ± 13	20.1 ± 9^a^	<0.001^†^	28 ± 15^ab^	18.5 ± 11^a^	17.7 ± 14^b^	<0.001^†^	31.2 ± 14^ab^	21.1 ± 12^a^	18.7 ± 12^b^	<0.001^†^
Current smoker (%)	5.6(4)	0	0	0.06	6.9(2)	5.6(7)	6.7(2)	0.94	7(7)	3.2(8)	3.9(2)	0.50
University graduate (%)	63.4(45)	72.4(92)	61.9(13)	0.33	69(20)	54.8(68)	60(18)	0.37	65(65)	63.5(160)	60.8(31)	0.87
Occupation *unemployed*	38(27)	37(47)	61.9(13)	0.08	44.8(13)	50(62)	46.7(14)	0.98	40(40)	43.7(110)	52.9(27)	0.48
*GE*	23.9(17)	37(47)	28.6(6)		27.6(8)	22.6(28)	26.7(8)		25(25)	29.8(75)	27.5(14)	
*worker*	11.3(8)	6.3(8)	0		3.4(1)	4.8(6)	6.7(2)		9(9)	5.6(14)	3.9(2)	
*Self-employment*	26.8(19)	19.7(25)	9.5(2)		24.1(7)	22.6(28)	20(6)		26(26)	21(53)	15.7(8)	

*GE* Government employee, *MET* Metabolic equivalents

Values are presented as mean ± standard deviation or numbers (%)

^*^ *P* values were obtained from ANOVA or χ2 tests, where appropriate. † *P* values were obtained from kruskal–wallis test. a & b Significant difference between genotype by ANOVA with Tukey’s post hoc tests

### Behavioral and hormonal parameters according to MC4R genotypes

Table 2 shows a comparison between behavioral and hormonal variables in different genotypes of MC4R rs17782313. A statistically significant difference was found in mean score of emotional eating between different genotypes of MC4R rs17782313 in individuals with obesity and all study participants (*p* ˂0.001).

**Table 2:** Association of MC4R variant rs17782313 with baseline behavior and biochemical characteristics

Variables	Overweight (*n* = 221)				Obese (*n* = 182)					Total (*n* = 403)				
	Genotype *P*-value^*^				Genotype *P*-value^*^					Genotype *P*-value^*^				
	TT(*n* = 71)	CT(*n* = 127)	CC(*n* = 23)		TT(*n* = 29)	CT(*n* = 123)	CC(*n* = 30)			TT(*n* = 100)	CT(*n* = 250)	CC(*n* = 53)		
Emotional eating (score)	9.3 ± 3.8	10.3 ± 5	11.8 ± 4	0.07	9.6 ± 4.8^ab^	13 ± 4.5^a^	14.2 ± 4^b^	<0.001		9.4 ± 4.1^ab^	11.6 ± 4.9^a^		13.2 ± 4.6^b^	<0.001
Depression (score)	3 ± 2.4^ab^	4.6 ± 3.2^a^	4.4 ± 2.8^b^	0.002	3.4 ± 2.9^ab^	4.7 ± 3^a^	6 ± 3.7^b^	0.008		3.2 ± 2.6^ab^	4.6 ± 3.1^a^		5.4 ± 3.4^b^	<0.001
Anxiety (score)	3.2 ± 1.7	3.2 ± 2	3.7 ± 2	0.57	3.3 ± 2.1	3.3 ± 1.8	3.3 ± 1.8	0.98		3.3 ± 1.8	3.4 ± 1.9		3.4 ± 1.9	0.81
Stress (score)	6.5 ± 3.6^ab^	7.6 ± 3.7^a^	8.9 ± 3.8^b^	0.04	5.3 ± 2.7^a^	7.1 ± 4.5^b^	7.5 ± 4.4^ab^	0.007		5.7 ± 3^ab^	7.4 ± 4.1^a^		8.3 ± 4^b^	<0.001
VAS (score)	50.3 ± 16^ab^	37.7 ± 20^a^	27.8 ± 18^b^	<0.001	44.9 ± 21^ab^	27.6 ± 19^a^	16 ± 15^b^	<0.001		48.8 ± 18^ab^	32.6 ± 20^a^		20.8 ± 18^b^	<0.001
Ghrelin (ng/ml)	0.57 ± 0.3^ab^	0.88 ± 0.3^a^	0.98 ± 0.5^b^	0.02^**^	0.76 ± 0.3^a^	0.8 ± 0.3	1.2 ± 0.8^a^	0.02^**^		0.68 ± 0.3^a^	0.85 ± 0.3		1.15 ± 0.7^a^	0.001^**^
GLP-1(pg/ml)	53.3 ± 20	52.3 ± 21	40 ± 18	0.17	59.2 ± 17^a^	50.5 ± 19	41.8 ± 18^a^	0.04		56.5 ± 18	47.7 ± 20		45.9 ± 19	0.08
Cortisol (ng/ml)	259 ± 153	335 ± 108	293 ± 122	0.26	320 ± 109^a^	318 ± 94^b^	354 ± 164^ab^	0.002		292 ± 133	328 ± 101		316 ± 147	0.54
Energy (kcal/d)	1877 ± 216	1914 ± 346	1930 ± 221	0.19^†^	2519 ± 341^a^	2550 ± 363^b^	2827 ± 452^ab^	0.01^†^		2098 ± 454^a^	2227 ± 423		2318 ± 525^a^	0.007^†^
Carbohydrate (kcal/d)	327 ± 40	322 ± 30	311 ± 25	0.14^†^	335 ± 56	320 ± 43	309 ± 39	0.08^†^		329 ± 45^a^	321 ± 37		309 ± 34^a^	0.01^†^
Protein (kcal/d)	87.1 ± 13	85.3 ± 12	83.1 ± 12	0.16^†^	88 ± 27	82 ± 19	73 ± 18	0.058^†^		86.1 ± 18^a^	82.4 ± 16		79.3 ± 17^a^	0.04^†^
Fat (kcal/d)	62.8 ± 13^ab^	73.9 ± 17^a^	80.6 ± 18^b^	0.002^†^	67.7 ± 10^ab^	73.1 ± 15^a^	81.2 ± 20^b^	<0.001^†^		66.3 ± 11^ab^	73.6 ± 16^a^		80.8 ± 18^b^	<0.001^†^

*VAS* Visual Analog Scale. Values are presented as mean ± standard deviation. * *P* values were obtained from ANOVA tests. **P values were obtained from kruskal–wallis test. †All values were adjusted for age, sex and energy (residual method), except for dietary energy intake, which was only adjusted for age and sex using ANCOVA. a & b Significant difference between genotype by ANOVA with Tukey’s post hoc test

No statistically significant difference was found between anxiety and different genotypes of MC4R rs17782313. However, there was a significant difference between depression and stress and MC4R rs17782313 genotypes in all study groups (*p* ˂0.05). TT genotype experienced lower level of stress and depression than C-allele carriers (Table 2).

Appetite of TT carriers was significantly lower than CT and CC carriers and C-allele carriers acquired the lowest score in visual analogue scale indicating increased appetite of these people (*p* ˂0.001) (Table 2).

A statistically significant difference was found in the level of ghrelin in all groups (*p* ˂0.05). There was also a statistically significant difference between GLP-1 (*p* = 0.03) and cortisol levels (*p* = 0.002) in different genotypes of MC4R rs17782313 in individuals with obesity (Table 2).

A statistically significant difference was found between mean intake of energy and different genotypes of MC4R rs17782313 in individuals with obesity (*p* = 0.01) and all participants (*p* = 0.007). CC genotype carriers had higher intake of energy than TT carriers. A statistically significant difference was also found between mean intake of carbohydrate and different genotypes of MC4R rs17782313 in all subjects (*p* = 0.01) (Table 2).

A statistically significant difference was found between mean intake of protein and different genotypes of MC4R rs17782313 in total subjects (*p* = 0.04). There was a statistically significant difference between mean intake of fat and different genotypes of MC4R rs17782313 in all study groups (*p* ˂0.001). TT genotype carriers had lower intake of fat than CC and CT genotype carriers (Table 2).

### Association between MC4R rs17782313 and food intake, Behavioral and hormonal parameters

Table 3 shows multivariate linear regression model of energy intake in different genotypes of MC4R rs17782313. Energy intake was found to be associated with the C allele in the total sample population. However, after stratification of the population into overweight and obese the results remained significant in the obese individual (*p* ˂0.05). A significant difference was also found in carbohydrate intake of CC carriers compared to TT carriers in all study groups according to Model 2. Carbohydrate intake in CC carriers in overweight people, CC carriers in individuals with obesity, and CC and CT carriers in all participants was less than TT genotype carriers by (-18.69), (-27.3), (-21.13), and (-9.54) units respectively.

**Table 3:** Association of MC4R variant rs17782313 with macronutrient intakes and energy intake in general linear models

Group	Group	Genotype	Crude		Model 1		Model 2	
			β (CI)	P	β (CI)	P	β (CI)	P
Energy (kcal/d)	Overweight	CT/TT CC/TT	24.47 (-47.79,96.75( -46.71 (-167.8, 74.40)	0.50 0.44	6.80 (-72.31, 85.92) -32.47 (-162.8, 97.89)	0.86^†^ 0.62^†^	75.36 (-5.73, 156.4) 60.60 (-70.4, 191.6)	0.06^†^ 0.36^†^
	Obese	CT/TT CC/TT	-70.09 (-183.8,43.64) 382.91 (215.2, 547.1)	0.22 <0.001	-34.50 (-184.7, 115.7) 379.2 (217.4, 540.1)	0.65^†^ <0.001^†^	-29.53(-190.7, 131.6) 311.5 (211.9, 539.8)	0.71^†^ 0.002^†^
	Total	CT/TT CC/TT	54.88 (-35.97, 145.7) 127.56 (-4.35, 259.48)	0.23 0.058	138.1 (34.76, 241.4) 218.6 (68.49, 368.8)	0.009^†^ 0.004^†^	146.8 (35.15, 258.6) 242.9 (83.31, 402.5)	0.01^†^ 0.003^†^
Carbohydrate (g/d)	Overweight	CT/TT CC/TT	-1.19 (-10.42, 8.02) -12.94 (-28.31, 2.42)	0.79 0.09	-5.69 (-15.83, 4.44) -16.19 (-32.91, 0.52)	0.27 0.05	-7.28 (-18.4, 3.83) -18.69 (-36.55, -0.83)	0.19 0.04
	Obese	CT/TT CC/TT	-2.12 (-16.39, 12.14) -14.05 (-31.95, 3.84)	0.76 0.12	-16 (-34.66, 2.65) -26.88 (-50.86, -2.90)	0.09 0.02	-17.20 (-37.30, 2.88) -27.30 (-52.67, -1.94)	0.09 0.03
	Total	CT/TT CC/TT	-1.71 (-9.74, 6.32) -13.78 (-25.4, -2.16)	0.67 0.02	-8.47 (-17.7, 0.82) -19.98 (-33.5, -6.44)	0.07 0.004	-9.54 (-19.6, -1.57) -21.13 (-35.64, -6.63)	0.04 0.004
Protein (g/d)	Overweight	CT/TT CC/TT	-2.68 (-6.09, 0.72) 3.25 (-2.48, 8.99)	0.12 0.26	-3.18 (-6.65, 0.28) 3.63 (-2.11, 9.37)	0.07 0.21	-2.99 (-6.95, 0.96) 2.11 (-4.24, 8.47)	0.13 0.51
	Obese	CT/TT CC/TT	1.21 (-5.37, 7.81) -9.36 (-17.57, -1.14)	0.71 0.02	1.73 (-4.82, 8.31) -9.51 (-17.85, -1.17)	0.60 0.03	3.41 (-3.16, 10) -8.17 (-16.42, -0.03)	0.30 0.05
	Total	CT/TT CC/TT	-1.37 (-4.83, 2.08) -4.20 (-9.22, 0.82)	0.43 0.10	-4.03 (-8.04, -0.02) -7.14 (-12.98, -1.30)	0.04 0.01	-4.83 (-8.84, -0.82) -7.65 (-13.51, -1.79)	0.05 0.01
Fat (g/d)	Overweight	CT/TT CC/TT	2.33 (-1.76, 6.43) 9.96 (3.20, 16.72)	0.26 0.004	5.65 (1.31, 9.98) 14.16 (7.01, 21.32)	0.01 <0.001	5.02 (0.30, 9.74) 14.25 (6.66, 21.84)	0.03 <0.001
	Obese	CT/TT CC/TT	2.07 (-3.52, 7.67) 8.75 (1.79, 15.72)	0.46 0.01	10.56 (3.57, 17.55) 15.41 (6.43, 24.40)	0.003 0.001	11.36 (3.83, 18.88) 16.18 (6.68, 25.68)	0.003 0.001
	Total	CT/TT CC/TT	2.41 (-0.92, 5.75) 9.26 (4.48, 14.05)	0.15 <0.001	6.63 (2.92, 10.35) 13.12 (7.70, 18.53)	0.001 <0.001	7.05 (3.02,11.08) 13.92 (8.15, 19.69)	0.001 <0.001

Model 1: adjusted for sex, age and energy intake. Model 2: adjusted for sex, age, energy intake, marital status, education, occupation, PA and smoking status. †Model adjusted for sex, age (model 1) and sex, age, marital status, education, occupation, PA and smoking status (model 2)

According to Model 2, CC genotype carriers had a lower intake of protein than TT genotype carriers in the total sample population (β = -7.65; 95%CI: -13.51, -1.79; *p*: 0.01). However, according to Model 1, both CC and CT carriers showed a lower intake of protein than TT genotype carriers in the total sample population (Table 3).

The results of linear regression analysis showed direct and significant relationship of fat consumption with C allele carriers compared to TT carriers in all study groups in Model 2 (Table 3).

Linear regression model also showed the minor allele (C) carriers scored lower in visual analogue scale and had higher appetite than TT genotype carriers in all study groups (*p* ˂0.05) (Supplementary Table 1). After adjustment for confounders in Model 2, the multivariate linear regression model showed ghrelin hormone was found to be associated with the genotype CC in the total sample population (*p* = 0.002). However, after stratification of the population into overweight and obese the results remained significant in the obese individuals (*p* = 0.04). (Supplementary Table 1). The results of linear regression analysis showed a significant inverse relationship between GLP-1 hormone and CC carriers in the obese group in Model 2 (*p* = 0.04). A significant and positive relationship was found between CC carriers and cortisol level in individuals with obesity in Model 2 after adjustment for confounders (*p* = 0.04) (Supplementary Table 1).

The emotional eating behavior was found to be associated with the C allele in the total sample population (*p* ˂0.05). However, after stratification of the population into overweight and obese the results remained significant in the CC genotype obese individuals (OR: 1.07; 95%CI: 1.01, 1.34; *p:* 0.001) (Table 4).

**Table 4:** Association of MC4R variant rs17782313 with behavioral parameters

Variable	Group	Genotype	Crude			Model 1			Model 2		
			OR	95%CI	*p*-value	OR	95%CI	*p*-value	OR	95%CI	*P*-value
Emotional eating	Overweight	CT/TT CC/TT	0.63 0.46	(0.24, 1.60) (0.11, 1.23)	0.33 0.12	0.44 0.65	(0.16, 1.25) (0.24, 1.73)	0.12 0.39	0.77 0.71	(0.25, 2.33) (0.26, 1.91)	0.65 0.50
	Obese	CT/TT CC/TT	0.40 1.09	(0.13, 1.25) (1.02, 1.34)	0.11 0.02	0.38 1.07	(0.12, 1.20) (1.01, 1.28)	0.09 0.01	0.30 1.07	(0.08, 1.04) (1.01, 1.34)	0.05 0.001
	Total	CT/TT CC/TT	1.21 1.48	(1.09, 1.44) (1.24, 1.95)	0.001 0.03	1.21 1.48	(1.09, 1.46) (1.24, 1.97)	0.001 0.04	1.31 1.47	(1.13, 1.71) (1.23, 1.98)	0.001 0.04
Depression	Overweight	CT/TT CC/TT	0.41 1.67	(0.06, 2.69) (0.36, 7.76)	0.36 0.51	0.38 1.62	(0.05, 2.53) (0.34, 7.63)	0.34 0.50	0.45 1.63	(0.06, 3.37) (0.33, 8.01)	0.35 0.51
	Obese	CT/TT CC/TT	0.26 0.34	(0.06, 1.12) (0.13, 0.88)	0.07 0.02	0.25 0.34	(0.05, 1.09( (0.12, 0.89)	0.07 0.02	0.39 0.39	(0.08, 1.86) (0.14, 1.07)	0.08 0.01
	Total	CT/TT CC/TT	0.23 0.58	(0.08, 0.67) (0.27, 1.25)	0.007 0.16	0.25 0.60	(0.08, 0.73) (0.28, 1.29)	0.01 0.19	0.36 0.68	(0.11, 1.31) (0.31, 1.49)	0.08 0.33
Anxiety	Overweight	CT/TT CC/TT	0.88 0.99	(0.08, 8.95) (0.11, 8.67)	0.91 0.99	0.82 1.06	(0.07, 8.55) (0.11, 9.67)	0.86 0.95	0.48 0.52	(0.03, 6.66) (0.04, 5.74)	0.58 0.59
	Obese	CT/TT CC/TT	3.34 0.96	(0.32, 34.19) (0.10, 8.97)	0.30 0.97	3.13 0.89	(0.28, 34.59) (0.09, 8.52)	0.35 0.92	4.19 0.61	(0.25, 68.65) (0.05, 7.12)	0.31 0.69
	Total	CT/TT CC/TT	1.56 1.01	(0.30, 8.03) (0.21, 4.76)	0.59 0.98	1.54 1.07	(0.29, 8.20) (0.22, 5.09)	0.60 0.93	1.17 0.79	(0.19, 7.21) (0.15, 3.98)	0.85 0.77
Stress	Overweight	CT/TT CC/TT	0.08 0.80	(0.008, 1.07) (0.21, 3.05)	0.07 0.74	0.07 0.74	(0.007, 1.03) (0.19, 2.93)	0.06 0.67	0.06 0.66	(0.004, 1.01) (0.15, 2.94)	0.05 0.59
	Obese	CT/TT CC/TT	0.32 0.87	(0.03, 3.28) (0.22, 3.35)	0.33 0.84	0.20 0.75	(0.01, 2.34) (0.17, 3.32)	0.20 0.70	0.25 1.23	(0.01, 3.42) (1.07, 1.95)	0.30 0.04
	Total	CT/TT CC/TT	0.15 0.86	(0.03, 1.78) (0.33, 2.21)	0.07 0.75	0.14 0.83	(0.02, 1.18) (0.31, 2.17)	0.07 0.70	0.20 0.96	(0.03, 1.14) (0.35, 2.64)	0.08 0.95

Model 1: adjusted for sex, age and energy intake. Model 2: adjusted for sex, age, energy intake, marital status, education, occupation, smoking status and physical activity

In Table 4, after adjustment for confounding factors in model 2, there was a significant relationship between depression and CC genotype in obese subjects (OR: 0.39; 95%CI: 0.14, 1.07; *p*: 0.01). The results of logistic regression analysis showed no statistically significant relationship between anxiety and MC4R rs17782313 genotypes in all study groups. There was also a statistically significant difference between CC genotype and stress in individuals with obesity according to Model 2. Stress level of CC carriers was 23% higher than TT carriers in individuals with obesity (Table 4).

There was a significant and inverse relationship between CC and CT genotype and physical activity in all study groups (*p* ˂ 0.05) (Supplementary Table 2).

### Interaction between MC4R rs17782313 and behavioral and hormonal parameters to determine the risk of obesity

There was interaction between high energy intake and MC4R polymorphism in determining the risk of obesity after adjusting for confounders such as age, gender and physical activity (OR = 1.39, *p* = 0.01) (Table 5).

**Table 5:** Interaction between MC4R polymorphism and behavior and hormonal parameters on risk of obesity

Variable	TT ^a^	CT	P_Interaction_	CC	P_Interaction_
High energy intake	1	0.62(0.1, 3.73)^b^	0.60	1.39 (1.08, 12.3)^b^	0.01
High fat intake	1	3.25 (0.24, 43.6)	0.37	23.43 (3.27, 167.9)	0.002
High carbohydrate intake	1	0.96 (0.12, 7.21)	0.97	6.85 (0.29, 191.8)	0.23
High protein intake	1	0.17 (0.02, 0.53)	0.01	1.59 (0.13, 19.32)	0.71
High physical activity	1	0.14 (0.02, 0.45)^c^	0.04	0.15 (0.01, 2.13)^c^	0.16
High emotional eating	1	17.99 (0.96, 336.5)	0.053	8.82 (1.35, 57.2)	0.02
High appetite	1	0.59 (0.02, 14.97)	0.75	1.55 (1.17, 11.19)	0.04
High anxiety	1	0.86 (0.01, 61.9)	0.94	0.66 (0.2, 78.1)	0.51
High depression	1	6.07 (0.17, 20.9)	0.31	38.8 (0.64, 132.4)	0.08
High stress	1	1.98 (0.73, 5.3)	0.17	4.31 (1.06, 17.4)	0.04
High ghrelin	1	0.05 (0.001, 5.1)	0.2	1.26 (1.04, 7.1)	0.03
High GLP-1	1	17.72 (0.48, 649.2)	0.11	0.89 (0.02, 40.06)	0.95
High cortisol	1	0.19 (0.005, 7.1)	0.37	1.09 (1, 8.87)	0.04

Data are presented as Odds ratio and 95% confidence intervals after adjusted for age, gender, energy intake, PA

^a^ Reference group

^b^ adjusted for age, gender, PA

^c^ adjusted for age, gender, energy intake

There was a significant interaction between high fat intake and CC genotype. The interaction increased the risk of obesity by 23.43 in CC genotype (*p* = 0.002). However, high carbohydrate intake and MC4R rs17782313 were not risk factor for obesity (*p* ˃ 0.05). Interaction of high protein intake and CT genotype was a significant risk factor for obesity (*p* = 0.01). High protein intake reduced obesity risk by 17% in CT heterozygotes. Physical activity reduced obesity risk by 14% in CT heterozygotes (*p* = 0.04) (Table 5).

There was a significant interaction between emotional eating and MC4R rs17782313 (*p* = 0.02). CC genotypes were more likely to show emotional eating behavior, which increased obesity risk by 8.82 times. The interaction of depression and anxiety with MC4R rs17782313 was not significant (*p* ˃ 0.05) (Table 5). However, there was a significant interaction between stress and MC4R rs17782313 (*p* = 0.04). The risk of obesity was 4.31 times higher in CC genotype carriers who experienced high level stress. The interaction of CC genotype and appetite increased obesity risk by 55% (*p* = 0.04). Ghrelin and cortisol levels were significantly higher in different MC4R rs17782313 genotypes. High ghrelin level increased obesity risk in CC genotype by 26% (*p* = 0.03). In turn, high cortisol level increased obesity risk in CC genotype by 9% (*p* = 0.04). There was no significant interaction between low levels of GLP-1 and MC4R polymorphism (*p* ˃ 0.05) (Table 5).

## Discussion

**Fig. 1:**
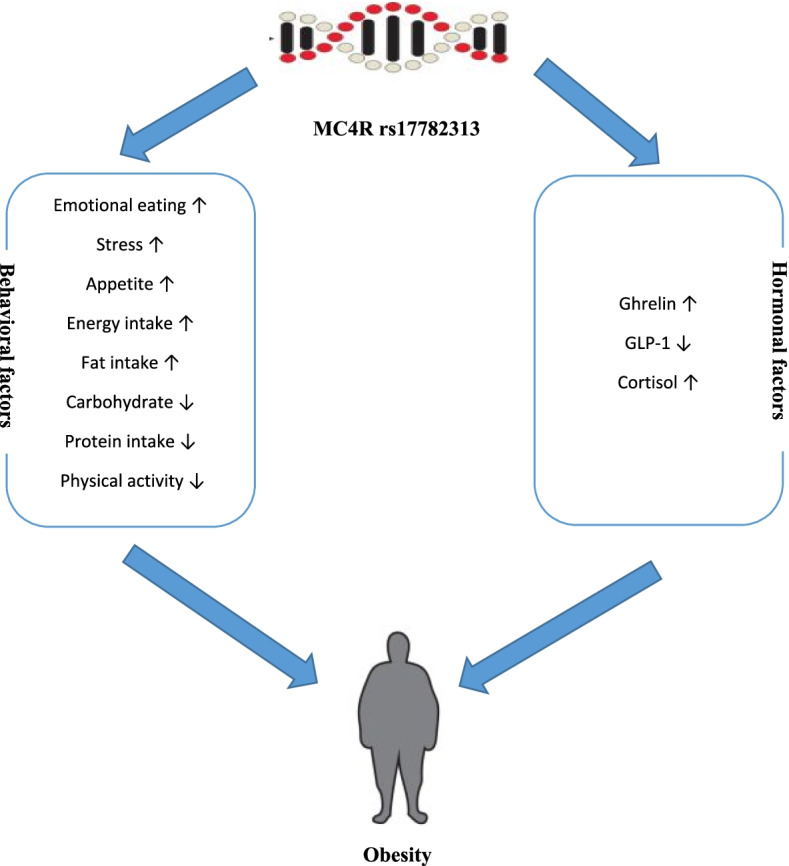
C allele of MC4R rs17782313 can modify the association between dietary intake, behavior and hormonal parameters and body weight

Obesity is mainly a complex disorder resulting from the interplay of environmental and genetic factors. Evidence suggests that MC4R polymorphism (rs17782313) is an important genetic factor in obesity that disrupts energy homeostasis [26]. This study aimed to assess the relationship of obesity-related biochemical and behavioral factors with rs17782313 variant in overweight and obese people and then assess the risk of obesity regarding interaction biochemical and behavioral factors with MC4R rs17782313 genotypes. The study's primary finding is that the MC4R SNP has the ability to alter the connection between food intake, behavior, hormonal markers, and body weight. Subjects with a high energy and fat intake, an increased appetite, stress, emotional eating, ghrelin, cortisol, and a low protein and physical activity intake, as well as the C allele of MC4R rs17782313, had a greater body weight (Fig. 1).

Minor allele frequency (MAF) of MC4R polymorphism (rs17782313) varied from 25% to 31.6% in some populations [12, 38–40]. However, findings of this study showed MAF was 44% of the Iranian population.

Khalilitehrani et al. [26] showed a higher intake of energy in CC genotype carriers, but a lower intake of protein and carbohydrates in CC genotype carriers than TT genotype carriers. These results were consistent with the results of this study. Khalilitehrani et al., on the other hand, discovered no link between fat intake and the MC4R rs17782313. These findings were not in line with the findings of this research. A large-scale cohort study showed that rs17782313 increased intake of fat and energy. The results of the former study showed that MC4R polymorphism is associated with a higher intake of protein [41]. These results were not consistent with the results of this study. Park et al. [42]. showed no significant difference between MC4R minor allele and energy intake. However, they showed higher fat intake in C-allele carriers than carriers of the major allele in this variant of MC4R gene. Different dietary assessment tools used in these studies might be the cause of these confounding results. Some studies showed that three-day food records showed greater agreement between observed and reported food intake than other standard dietary assessment methods, including 24- hour dietary recalls [43]. Recent studies suggested that the hypothalamic melanocortin system is responsible for the regulation of food intake and weight gain in case of the availability of different sources of macronutrients. Experimental study showed the upregulation of MC3R that compensate for the loss of MC4R function and allow the intake of other nutrients to maintain normal body weight [44]. Although experimental evidence suggests the association of rs17782313 with obesity in terms of higher intake of food, human studies showed conflicting relationships between rs17782313 variant and dietary intake and energy intake [45]. Nevertheless, many confounding variables, such as diverse populations with varied environmental and genetic characteristics, different dietary assessment methodologies, and inaccurate self-reporting of food consumption, might be the origin of these contradictory findings [46].

Experimental studies showed that loss of MC4R function is associated with overeating, hyperinsulinemia, and obesity. Despite similar food intake, these studies have shown that C-allele carriers gain more weight than homozygous dominant in mice. It was suggested that C-allele carriers had lower energy expenditure than energy intake and gained more weight [47]. These results were consistent with the results of this study. The results showed that defects in the melanocortin pathways decreased sympathetic stimulation and induction of lipolysis by sympathetic nerve activity [48]. Regular physical exercise seems to promote neurotransmitter activity, which in turn increases the release of monoamines like dopamine and norepinephrine. As a result, physical exercise may be able to compensate for anomalies in energy-regulating melanocortin pathways [14]. It was suggested that although two normal copies of MC4R gene were necessary for normal weight regulation, but single normal copy of this gene is needed for weight loss effects in case that gastric bypass surgery intervention was used to reduce weight gain [49]. Khalilitehrani et al. also found out that both intensity and length of physical activity were significantly correlated with BMI in heterozygotes in comparison with other genotypes [14]. Although some studies, including this study showed a significant relationship between physical activity and different MC4R rs17782313 genotypes [50], others showed no statistically significant relationship between these two factors [10, 39]. The necessity for MC4R signaling to impart the impact of physical activity on BMI might be one explanation for the association between physical activity and BMI in heterozygous people and the lack of this correlation in recessive homozygotes in our research. Nevertheless, the relationship observed in this study might be completely random because of scant literature on the subject and contradictory findings reported by previous studies. Hence, it is necessary to design further studies to reach a definite answer.

Many studies have reported the association between MC4R variants and obesity, especially rs17782313 [1, 51]. Appetite is the key to the interpretation of this association. Ghrelin and GLP-1 are both involved in the regulation of obesity [20, 22], which justifies different levels of these hormones in CC genotypes. Ghrelin has a well-established role in the hypothalamic regulation of appetite. This hormone reduces appetite by decreasing Pro-opiomelanocortin (POMC) neuronal activity and increasing AgRP neuronal activity [1]. GLP-1 suppresses hunger by activating the glucagon-like peptide 1 receptor (GLP-1R) in the peripheral and central nervous systems, resulting in weight loss [52]. MC4R in L cells stimulates the release of GLP-1 and Peptide YY (PYY) in the presence of melanocortin peptides [53]. Thus, mutations in MC4R can alter the secretion of GLP-1. The results of this study showed an association of high levels of ghrelin and low levels of GLP-1 with high levels of appetite in carriers of the minor allele, especially in obese people. High levels of appetite in carriers of CC genotype as well as high levels of ghrelin in carriers of CC genotype increased the risk of obesity. However, the results of some studies were consistent and some were not consistent with the results of this study. The results of a cohort study showed higher ghrelin levels in carriers of C-allele compared to T-allele carriers [1]. Magno et al*.* found out that carriers of C-allele in MC4R polymorphism had high plasma levels of ghrelin, but they found no difference in the appetite of their study groups [54]. Arizablaga et al*.* did not find any significant difference in ghrelin levels between C-allele and T-allele carriers in MC4R gene [55]. Different types of studies and populations might cause these confounding results.

There is not adequate information on the mechanism of MC4R rs17782313 and high BMI. Few studies have assessed the role of eating behavior in the association of MC4R rs17782313 with BMI. This study showed an association among minor allele carriers in all participants and CC genotype in obese people with emotional eating. The results showed that emotional eating behavior in CC genotype is a risk factor for obesity. This is an interesting finding. An imaging study offered preliminary evidence for the involvement of MC4R in emotional eating [56]. Yilmaz et al*.* showed that C-allele in rs17782313 variant was associated with increased emotional eating behaviors and food craving [57]. The association of MC4R with emotional eating in this study clarify MC4R-dopamine interaction [58]. Dopamine is overexpressed in reward pathways of the brain. Many studies reported the involvement of dopaminergic genes in binge eating [59, 60]. Many studies reported the association between dopamine receptor D2 (DRD2) markers and emotional eating [61].

Many studies showed that MC4R expression in the hypothalamus leads to excessive energy intake. Stress activates hypothalamic–pituitary–adrenal (HPA) axis that regulates energy balance [26]. This was the first study that assessed the interaction between MC4R rs17782313 variant and stress, anxiety, depression, and stress hormone (cortisol) affects obesity risk. A positive interaction was found between CC genotype of MC4R rs17782313 variant and stress and cortisol levels. Park et al*.* found out that the interaction of C-allele in MC4R gene with high stress was a risk factor for obesity [42]. There was no link found between depression, anxiety, or the CC genotype. Although the combination of depression and anxiety with the CC genotype was not a substantial risk factor for obesity, Yilmaz discovered a strong link between rs17782313 and depression [57]. Nevertheless, accumulating evidence in the last ten years showed that MC4R is involved in the regulation of anxiety and the neurobiology of depression. Some evidence emphasized the importance of melanocortin system (especially MC4R) in stress-induced changes, behavioral responses to stress, and stress-related disorders [62].

Acute stress activates POMC, which releases *α*-Melanocyte-stimulating hormone (α-MSH) that consequently increases MC4R levels. MC4R activates the HPA axis and adrenocorticotropic hormones (ACTH) which increase the release of cortisol in response to stress [63]. Various studies have shown that high-stress levels alter eating behaviors, which lead to increased intake of high-calorie junk food which increases the risk of overweight and obesity [64].

Several limitations of the current study should also be considered. First, Similar to Jeffery's study on interleukin-18 polymorphism, genotypes were not in Hardy–Weinberg equilibrium. This may have been due to the small sample size resulting from time and financial constraints. Therefore, future studies are recommended to work with larger sample sizes [65]. Second, this was a cross-sectional research that was unable to demonstrate a causal association between the variables evaluated. It just proposed a notion that will be tested in future investigations. The exclusion of individuals with normal BMI was another research limitation. Future studies can investigate the normal BMI individuals and their results can be compared with those of the present study. Underreporting of food intakes, which are typically seen in obese people, may contribute to bias and null findings [66]. However, subjects with extreme dietary intake values were not included in the analysis. On the other hand, current study as the same as other observational studies is prone to residual confounding in terms of unknown or unmeasured confounders [67]. Despite the limitations discussed above, this is the first attempt to study the association between MC4R rs17782313 polymorphisms and behavioral and hormonal factors in obese and overweight individuals and interaction among MC4R rs17782313 polymorphisms and food intake, stress, anxiety, depression, physical activity, emotional eating and appetite on obesity, according to our knowledge. Identifying these gene-behavioral factor interactions could be crucial in planning appropriate personalized nutritional advice for the prevention and management of obesity and its related consequences.

## Conclusion

In conclusion, this study for the first time produced initial evidence that there may be an interactive effect between food intake, behavioral and hormonal factors and the MC4R rs17782313 genotypes on BMI. We found that adherence to a high-energy and fat diet and low protein diet in subjects with the C allele of MC4R rs17782313 is related to a higher BMI. These data also emphasize that individuals with the C allele of rs17782313 in MC4R with a high-emotional eating, appetite, stress, cortisol, ghrelin and low physical activity had higher BMI; in fact, these subjects are more susceptible to being overweight/obese. Additional studies are needed by including the clinical level to clear up the biology of MC4R rs17782313 and its impact on the relationship between behavioral and hormonal factors and degree of obesity.

## Supplementary Information

**Additional file 1: Supplementary Table 1.** Association of MC4R variant rs17782313 with appetite and biochemical parameters in general linear models. **Supplementary Table 2.** Association of MC4R variant rs17782313 with physical activity in general linear models.

## Data Availability

The related data of studied MC4R variant is deposited in dbSNP database and accessible via rs17782313 accession number, https://www.ncbi.nlm.nih.gov/snp/rs17782313. Moreover, the flanking sequence harboring rs17782313 variant was extracted from NM_005912.3 transcript.

## Abbreviations

ACTH: Adrenocorticotropic hormones
AgRP: Agouti-related protein
ANCOVA: Analysis of covariance
CNS: Central nervous system
CI: Confidence intervals
DASS-21: Depression, anxiety, stress scale-21
DRD2: Dopamine receptor D2
EEQ: Emotional Eating Questionnaire
EDTA: Ethylenediaminetetraacetic acid
FCT: Food Composition Table
GLP-1R: Glucagon-like peptide 1 receptor
GLP-1: Glucagon-like peptide-1
HC: Hip circumference
HPA: Hypothalamic–pituitary–adrenal
IPAQ: International Physical Activity Questionnaire
MC4R: Melanocortin-4 receptor
MAF: Minor allele frequency
OR: Odds ratio
PYY: Peptide YY
PA: Physical activity
PCR: Polymerase Chain Reaction
POMC: Pro-opiomelanocortin
RFLP: Restriction Fragment Length Polymorphism
SD: Standard deviation
VAS: Visual analogue scales
WC: Waist circumference
WHR: Waist-to-hip ratio
α-MSH: *α*-Melanocyte-stimulating hormone

## References

[1] Hammad MM, Abu-Farha M, Hebbar P, Cherian P, Al Khairi I, Melhem M (2020). MC4R Variant rs17782313 associates with increased levels of DNAJC27, ghrelin, and visfatin and correlates with obesity and hypertension in a Kuwaiti cohort. Front Endocrinol.

[2] Balthasar N, Dalgaard LT, Lee CE, Yu J, Funahashi H, Williams T (2005). Divergence of melanocortin pathways in the control of food intake and energy expenditure. Cell.

[3] Podyma B, Sun H, Wilson EA, Carlson B, Pritikin E, Gavrilova O (2018). The stimulatory G protein Gsα is required in melanocortin 4 receptor–expressing cells for normal energy balance, thermogenesis, and glucose metabolism. J Biol Chem.

[4] Razquin C, Marti A, Martinez JA (2011). Evidences on three relevant obesogenes: MC4R, FTO and PPARγ. approaches for personalized nutrition. Mol Nutr Food Res.

[5] Ghamari-Langroudi M, Digby GJ, Sebag JA, Millhauser GL, Palomino R, Matthews R (2015). G-protein-independent coupling of MC4R to Kir7. 1 in hypothalamic neurons. Nature..

[6] Kohlsdorf K, Nunziata A, Funcke J-B, Brandt S, von Schnurbein J, Vollbach H (2018). Early childhood BMI trajectories in monogenic obesity due to leptin, leptin receptor, and melanocortin 4 receptor deficiency. Int J Obes.

[7] Berglund ED, Liu T, Kong X, Sohn J-W, Vong L, Deng Z (2014). Melanocortin 4 receptors in autonomic neurons regulate thermogenesis and glycemia. Nat Neurosci.

[8] Muller YL, Thearle MS, Piaggi P, Hanson RL, Hoffman D, Gene B (2014). Common genetic variation in and near the melanocortin 4 receptor gene (MC4R) is associated with body mass index in American Indian adults and children. Hum Genet.

[9] Beckers S, Zegers D, de Freitas F, Mertens IL, Van Gaal LF, Van Hul W (2011). Association study of MC4R with complex obesity and replication of the rs17782313 association signal. Mol Genet Metab.

[10] Corella D, Ortega-Azorín C, Sorlí JV, Covas MI, Carrasco P, Salas-Salvadó J (2012). Statistical and biological gene-lifestyle interactions of MC4R and FTO with diet and physical activity on obesity: new effects on alcohol consumption. PLoS ONE.

[11] Martins MC, Trujillo J, Freitas-Vilela AA, Farias DR, Rosado EL, Struchiner CJ (2018). Associations between obesity candidate gene polymorphisms (fat mass and obesity-associated (FTO), melanocortin-4 receptor (MC4R), leptin (LEP) and leptin receptor (LEPR)) and dietary intake in pregnant women. Br J Nutr.

[12] Valette M, Bellisle F, Carette C, Poitou C, Dubern B, Paradis G (2013). Eating behaviour in obese patients with melanocortin-4 receptor mutations: a literature review. Int J Obes.

[13] Alizadeh S, Pooyan S, Mirzababaei A, Arghavani H, Hasani H, Mirzaei K (2020). Interaction of MC4R rs17782313 variants and dietary carbohydrate quantity and quality on basal metabolic rate and general and central obesity in overweight/obese women: a cross-sectional study. BMC Endocr Disord..

[14] Khalilitehrani A, Ghorbani M, Hosseini S, Pishva H (2015). Association between physical activity and body mass index in different melanocortin receptor 4 (MC4R) genotypes. J Public Health Res.

[15] Stutzmann F, Cauchi S, Durand E, Calvacanti-Proenca C, Pigeyre M, Hartikainen A (2009). Common genetic variation near MC4R is associated with eating behaviour patterns in European populations. Int J Obes.

[16] Chaffin AT, Fang Y, Larson KR, Mul JD, Ryan KK (2019). Sex-dependent effects of MC4R genotype on HPA axis tone: implications for stress-associated cardiometabolic disease. Stress.

[17] Ulrich-Lai YM, Fulton S, Wilson M, Petrovich G, Rinaman L (2015). Stress exposure, food intake and emotional state. Stress.

[18] Wu Y-K, Brownley KA, Bardone-Cone AM, Bulik CM, Baker JH (2021). Associations of stress and appetite hormones with binge eating in females with anorexia nervosa after weight restoration: a longitudinal study. J Pers Med.

[19] Chao AM, Jastreboff AM, White MA, Grilo CM, Sinha R (2017). Stress, cortisol, and other appetite-related hormones: Prospective prediction of 6-month changes in food cravings and weight. Obesity.

[20] Grill HJ (2020). A role for GLP-1 in treating hyperphagia and obesity. Endocrinology..

[21] Krieger J-P (2020). Intestinal glucagon-like peptide-1 effects on food intake: physiological relevance and emerging mechanisms. Peptides.

[22] Di Bonaventura EM, Botticelli L, Del Bello F, Giorgioni G, Piergentili A, Quaglia W (2021). Assessing the role of ghrelin and the enzyme ghrelin O-acyltransferase (GOAT) system in food reward, food motivation, and binge eating behavior. Pharmacol Res.

[23] Al Massadi O, López M, Tschöp M, Diéguez C, Nogueiras R (2017). Current understanding of the hypothalamic ghrelin pathways inducing appetite and adiposity. Trends Neurosci.

[24] Evers C, Marijn Stok F, de Ridder DT (2010). Feeding your feelings: emotion regulation strategies and emotional eating. Pers Soc Psychol Bull.

[25] López-Guimerà G, Dashti HS, Smith CE, Sánchez-Carracedo D, Ordovas JM, Garaulet M (2014). CLOCK 3111 T/C SNP interacts with emotional eating behavior for weight-loss in a Mediterranean population. PLoS ONE.

[26] Khalilitehrani A, Qorbani M, Hosseini S, Pishva H (2015). The association of MC4R rs17782313 polymorphism with dietary intake in Iranian adults. Gene.

[27] Zlatohlavek L, Vrablik M, Motykova E, Ceska R, Vasickova L, Dlouha D (2013). FTO and MC4R gene variants determine BMI changes in children after intensive lifestyle intervention. Clin Biochem.

[28] Zaremba SM, Gow IF, Drummond S, McCluskey JT, Steinert RE (2018). Effects of oat β-glucan consumption at breakfast on ad libitum eating, appetite, glycemia, insulinemia and GLP-1 concentrations in healthy subjects. Appetite.

[29] Akamizu T, Shinomiya T, Irako T, Fukunaga M, Nakai Y, Nakai Y (2005). Separate measurement of plasma levels of acylated and desacyl ghrelin in healthy subjects using a new direct ELISA assay. J Clin Endocrinol Metab.

[30] Shelton MM, Schminkey DL, Groer MW (2015). Relationships among prenatal depression, plasma cortisol, and inflammatory cytokines. Biol Res Nurs.

[31] Organization WHOJGWH (1989). Measuring obesity: classification and description of anthropometric data.

[32] Vasheghani-Farahani A, Tahmasbi M, Asheri H, Ashraf H, Nedjat S, Kordi R (2011). The Persian, last 7-day, long form of the international physical activity questionnaire: translation and validation study. Asian J Sports Med.

[33] Committee IR. Guidelines for data processing and analysis of the International Physical Activity Questionnaire (IPAQ)-short and long forms.CiNii Article. 2005. http://www.ipaq.ki.se/scoring.pdf.

[34] Gibbons C, Hopkins M, Beaulieu K, Oustric P, Blundell JE (2019). Issues in measuring and interpreting human appetite (satiety/satiation) and its contribution to obesity. Curr Obes Rep.

[35] Garaulet M, Canteras M, Morales E, López-Guimerà G, Sánchez-Carracedo D, Corbalán-Tutau M (2012). Validation of a questionnaire on emotional eating for use in cases of obesity; the Emotional Eater Questionnaire (EEQ). Nutr Hosp.

[36] Henry JD, Crawford JR (2005). The short-form version of the Depression Anxiety Stress Scales (DASS-21): construct validity and normative data in a large non-clinical sample. Br J Clin Psychol.

[37] Moghaddam A, Saed F, Dibajnia P, Zangeneh J (2008). A preliminary validation of the depression, anxiety and stress scales (DASS) in non-clinical sample. Clin Psychol Personal.

[38] Bauer F, Elbers CC, Adan RA, Loos RJ, Onland-Moret NC, Grobbee DE (2009). Obesity genes identified in genome-wide association studies are associated with adiposity measures and potentially with nutrient-specific food preference. Am J Clin Nutr.

[39] Liem ET, Vonk JM, Sauer PJ, van der Steege G, Oosterom E, Stolk RP (2010). Influence of common variants near INSIG2, in FTO, and near MC4R genes on overweight and the metabolic profile in adolescence: the TRAILS (TRacking Adolescents’ Individual Lives Survey) Study. Am J Clin Nutr.

[40] Liu G, Zhu H, Dong Y, Podolsky RH, Treiber FA, Snieder H (2011). Influence of common variants in FTO and near INSIG2 and MC4R on growth curves for adiposity in African–and European-American youth. Eur J Epidemiol.

[41] Qi L, Kraft P, Hunter DJ, Hu FB (2008). The common obesity variant near MC4R gene is associated with higher intakes of total energy and dietary fat, weight change and diabetes risk in women. Hum Mol Genet.

[42] Park S, Daily JW, Zhang X, Jin HS, Lee HJ, Lee YH (2016). Interactions with the MC4R rs17782313 variant, mental stress and energy intake and the risk of obesity in Genome Epidemiology Study. Nutr Metab.

[43] Crawford PB, Obarzanek E, Morrison J, Sabry Z (1994). Comparative advantage of 3-day food records over 24-hour recall and 5-day food frequency validated by observation of 9-and 10-year-old girls. J Am Diet Assoc.

[44] Pillot B, Duraffourd C, Bégeot M, Joly A, Luquet S, Houberdon I (2011). Role of hypothalamic melanocortin system in adaptation of food intake to food protein increase in mice. PLoS ONE.

[45] Hasselbalch AL, Angquist L, Christiansen L, Heitmann BL, Kyvik KO, Sørensen TI (2010). A variant in the fat mass and obesity-associated gene (FTO) and variants near the melanocortin-4 receptor gene (MC4R) do not influence dietary intake. J Nutr.

[46] Carroll RJ, Midthune D, Subar AF, Shumakovich M, Freedman LS, Thompson FE (2012). Taking advantage of the strengths of 2 different dietary assessment instruments to improve intake estimates for nutritional epidemiology. Am J Epidemiol.

[47] Hinney A, Volckmar A-L, Knoll N (2013). Melanocortin-4 receptor in energy homeostasis and obesity pathogenesis. Prog Mol Biol Transl Sci.

[48] Sayk F, Heutling D, Dodt C, Iwen KA, Wellhoner JP, Scherag S (2010). Sympathetic function in human carriers of melanocortin-4 receptor gene mutations. J Clin Endocrinol Metab.

[49] Hatoum IJ, Stylopoulos N, Vanhoose AM, Boyd KL, Yin DP, Ellacott KL (2012). Melanocortin-4 receptor signaling is required for weight loss after gastric bypass surgery. J Clin Endocrinol Metab.

[50] Xi B, Wang C, Wu L, Zhang M, Shen Y, Zhao X (2011). Influence of physical inactivity on associations between single nucleotide polymorphisms and genetic predisposition to childhood obesity. Am J Epidemiol.

[51] Graff M, Scott RA, Justice AE, Young KL, Feitosa MF, Barata L (2017). Genome-wide physical activity interactions in adiposity-A meta-analysis of 200,452 adults. PLoS Genet.

[52] Iepsen EW, Zhang J, Thomsen HS, Hansen EL, Hollensted M, Madsbad S (2018). Patients with obesity caused by melanocortin-4 receptor mutations can be treated with a glucagon-like peptide-1 receptor agonist. Cell Metab.

[53] Panaro BL, Tough IR, Engelstoft MS, Matthews RT, Digby GJ, Møller CL (2014). The melanocortin-4 receptor is expressed in enteroendocrine L cells and regulates the release of peptide YY and glucagon-like peptide 1 in vivo. Cell Metab.

[54] Magno FCCM, Guaraná HC, da Fonseca ACP, Pedrosa AP, Zembrzuski VM, Cabello PH (2021). Association of the MC4R rs17782313 polymorphism with plasma ghrelin, leptin, IL6 and TNFα concentrations, food intake and eating behaviors in morbidly obese women. Eat Weight Disord.

[55] Arrizabalaga M, Larrarte E, Margareto J, Maldonado-Martín S, Barrenechea L, Labayen I (2014). Preliminary findings on the influence of FTO rs9939609 and MC4R rs17782313 polymorphisms on resting energy expenditure, leptin and thyrotropin levels in obese non-morbid premenopausal women. J Physiol Biochem.

[56] Horstmann A, Kovacs P, Kabisch S, Boettcher Y, Schloegl H, Tönjes A (2013). Common genetic variation near MC4R has a sex-specific impact on human brain structure and eating behavior. PLoS ONE.

[57] Yilmaz Z, Davis C, Loxton NJ, Kaplan AS, Levitan RD, Carter JC (2015). Association between MC4R rs17782313 polymorphism and overeating behaviors. Int J Obes.

[58] Pandit R, Van Der Zwaal EM, Luijendijk MC, Brans MA, Van Rozen AJ, Oude Ophuis RJ (2015). Central melanocortins regulate the motivation for sucrose reward. PLoS ONE.

[59] Levitan RD, Masellis M, Basile VS, Lam RW, Kaplan AS, Davis C (2004). The dopamine-4 receptor gene associated with binge eating and weight gain in women with seasonal affective disorder: an evolutionary perspective. Biol Psychiat.

[60] Davis JF, Choi DL, Shurdak JD, Krause EG, Fitzgerald MF, Lipton JW (2011). Central melanocortins modulate mesocorticolimbic activity and food seeking behavior in the rat. Physiol Behav.

[61] Davis C, Levitan RD, Yilmaz Z, Kaplan AS, Carter JC, Kennedy JL (2012). Binge eating disorder and the dopamine D2 receptor: genotypes and sub-phenotypes. Prog Neuropsychopharmacol Biol Psychiatry.

[62] Serova LI, Laukova M, Alaluf LG, Sabban EL (2013). Intranasal infusion of melanocortin receptor four (MC4R) antagonist to rats ameliorates development of depression and anxiety related symptoms induced by single prolonged stress. Behav Brain Res.

[63] Liu J, Garza JC, Li W, Lu X-Y (2013). Melanocortin-4 receptor in the medial amygdala regulates emotional stress-induced anxiety-like behaviour, anorexia and corticosterone secretion. Int J Neuropsychopharmacol.

[64] Sinha R, Jastreboff AM (2013). Stress as a common risk factor for obesity and addiction. Biol Psychiat.

[65] Szeszko JS, Howson JM, Cooper JD, Walker NM, Twells RC, Stevens HE (2006). Analysis of polymorphisms of the interleukin-18 gene in type 1 diabetes and Hardy-Weinberg equilibrium testing. Diabetes.

[66] Fisher JO, Johnson RK, Lindquist C, Birch LL, Goran MI (2000). Influence of body composition on the accuracy of reported energy intake in children. Obes Res.

[67] Nørgaard M, Ehrenstein V, Vandenbroucke JP (2017). Confounding in observational studies based on large health care databases: problems and potential solutions–a primer for the clinician. Clin Epidemiol.

