# Supplementary material for: Association and interaction of the MC4R rs17782313 polymorphism with plasma ghrelin, GLP-1, cortisol, food intake and eating behaviors in overweight/obese Iranian adults
Source: BMC Endocr Disord. 2022 Sep 19;22:234. doi: 10.1186/s12902-022-01129-w (PMC9487018; doi:10.1186/s12902-022-01129-w)
Supplement: Supplementary file 1 — Additional file 1: Supplementary Table 1. Association of MC4R variant rs17782313 with appetite and biochemical parameters in general linear models. Supplementary Table 2. Association of MC4R variant rs17782313 with physical activity in general linear models. [file 12902_2022_1129_MOESM1_ESM.docx]

| Group | Group | Genotype | | Crude |  | Model 1 |  | Model 2 |  |
| --- | --- | --- | --- | --- | --- | --- | --- | --- | --- |
|  |  |  | | β (CI) | P | β (CI) | P | β (CI) | P |
| VAS. score | Overweight | CT/TT  CC/TT | | -7.52 (-12.90, -2.15)  -14.40 (-23.37, -5.43) | 0.006  0.002 | -12.87 (-18.48, -7.26)  -22.03 (-31.29, -12.78) | ˂0.001  ˂0.001 | -9.70 (-15.63, -3.77)  -18.25 (-27.78, -8.72) | 0.001  ˂0.001 |
|  | Obese | CT/TT  CC/TT | | -2.59 (-9.19, 4.00)  -14.89 (-22.95, -6.84) | 0.44  ˂0.001 | -17.88 (-25.76, -10.01)  -28.41 (-38.54, -18,29) | ˂0.001  ˂0.001 | -13.11 (-21.24, -4.97)  -23.66 (-33.93,-13.38) | 0.002  ˂0.001 |
|  | Total | CT/TT  CC/TT | | -6.75 (-11.06, -2.43)  -16.33 (-22.48, -10.18) | 0.002  ˂0.001 | -15.05 (-19.55, -10.56)  -26.07 (-32.61, -19.53) | ˂0.001  ˂0.001 | -10.45 (-15.14, -5.77)  -21.04 (-27.74, -14.34) | ˂0.001  ˂0.001 |
| Ghrelin (ng/ml) | Overweight | CT/TT  CC/TT | | 0.10 (-0.15, 0.37)  0.23 (-0.03, 0.51) | 0.41  0.08 | 0.26 (-0.03, 0.57)  0.36 (0.03, 0.68) | 0.08  0.03 | 0.14 (-0.20, 0.48)  0.28 (-0.08, 0.65) | 0.40  0.12 |
|  | Obese | CT/TT  CC/TT | | -0.22 (-0.61, 0.16)  0.49 (0.15, 0.83) | 0.25  0.005 | 0.14 (-0.27, 0.56)  0.52 (0.12, 0.93) | 0.48  0.01 | 0.11 (-0.32, 0.55)  0.44 (0.01, 0.88) | 0.58  0.04 |
|  | Total | CT/TT  CC/TT | | -0.06 (-0.30, 0.16)  0.38 (0.16, 0.60) | 0.57  0.001 | 0.22 (-0.02, 0.47)  0.48 (0.24, 0.73) | 0.07  ˂0.001 | 0.15 (-0.10, 0.40)  0.41 (0.15, 0.68) | 0.23  0.002 |
| GLP-1(pg/ml) | Overweight | CT/TT  CC/TT | | 5.37 (-7.54, 18.29)  -12.7 (-26.03, 0.62) | 0.40  0.06 | -3.02 (-17.8, 11.8)  -16.3 (-32.3, -0.41) | 0.68  0.04 | -2.45 (-17.2, 9.4)  -11.2 (-29.7, 7.32) | 0.57  0.22 |
|  | Obese | CT/TT  CC/TT | | -0.93 (-12.9, 11.0)  -12.93 (-25.2, -0.66) | 0.87  0.03 | -6.35 (-20.8, 8.11)  -16.5 (-31.4, -1.62) | 0.38  0.03 | -3.67 (-18.8, 11.4)  -15.2 (-30.4, -0.04) | 0.62  0.04 |
|  | Total | CT/TT  CC/TT | | -3.47 (-12.32, 5.3)  -6.14 (-14.93, 2.63) | 0.43  0.16 | -8.36 (-18.51, 1.79)  -9.68 (-19.76, 0.40) | 0.10  0.06 | -6.60 (-17.12, 3.91)  -5.89 (-16.81, 5.02) | 0.21  0.28 |
| Cortisol (ng/ml) | Overweight | CT/TT  CC/TT | | 59.71 (-19.9, 139.4)  -8.21 (-95.5, 79.1) | 0.13  0.85 | 81.06 (-15.7, 177.8)  43.28 (-60.7, 147.3) | 0.09  0.40 | 82.37 (-18.1, 182.9)  53.76 (-54.4, 161.9) | 0.10  0.31 |
|  | Obese | CT/TT  CC/TT | -9.72 (-94.24, 74.79)  15.45 (-63.2, 94.2) | | 0.81  0.69 | 26.88 (-62.9, 116.6)  35.86 (-51.3, 123.1) | 0.54  0.41 | -2.92 (-92.1, 86.2)  5.36 (0.01, 33.4) | 0.94  0.04 |
|  | Total | CT/TT  CC/TT | 23.59 (-33.4, 80.5)  6.69 (-50.4, 63.8) | | 0.41  0.81 | 50.64 (-14.8, 116.1)  29.52 (-35.4, 94.5) | 0.12  0.36 | 25.81 (-41.3, 92.9)  -4.12 (-73.7, 65.5) | 0.44  0.90 |

Supplementary Table 1. Association of MC4R variant rs17782313 with appetite and biochemical parameters in general linear models.

Model 1: adjusted for sex, age, energy intake. Model 2: adjusted for sex, age, energy intake, marital status, education, occupation, physical activity and smoking status.

| Group | Group | Genotype | Crude | |  | Model 1 | |  | Model 2 | |  |
| --- | --- | --- | --- | --- | --- | --- | --- | --- | --- | --- | --- |
|  |  |  | β | CI | P | β | CI | P | β | CI | P |
| Physical activity  (met-h/week) | Overweight | CT/TT  CC/TT | -5.87  -6.73 | -9.59, -2.16  -13.0, -0.43 | 0.002  0.03 | -9.92  -11.6 | -13.5, -6.3  -17.5, -5.6 | ˂0.001  ˂0.001 | -9.91  -12 | -13.6, -6.2  -18.1, -5.9 | ˂0.001  ˂0.001 |
|  | Obese | CT/TT  CC/TT | -4.45  -2.62 | -8.44, -0.47  -7.70, 2.45 | 0.02  0.30 | -10.4  -12 | -15.4, -5.4  -18.5, -5.6 | ˂0.001  ˂0.001 | -11  -11.6 | -15.8, -6.2  -17.7, -5.4 | ˂0.001  ˂0.001 |
|  | Total | CT/TT  CC/TT | -5.92  -5.24 | -8.67, -3.17  -9.30, -1.18 | ˂0.001  0.01 | -11.0  -13.5 | -14.0, -8.10  -17.8, -9.21 | ˂0.001  ˂0.001 | -11.3  -13.6 | -14.3, -8.3  -18.0, -9.3 | ˂0.001  ˂0.001 |

Supplementary Table 2. Association of MC4R variant rs17782313 with physical activity in general linear models.

Model 1: adjusted for sex, age and energy intake. Model 2: adjusted for sex, age, energy intake, marital status, education, occupation and smoking status.
